# Potential of Bone-Marrow-Derived Mesenchymal Stem Cells for Maxillofacial and Periodontal Regeneration: A Narrative Review

**DOI:** 10.1155/2021/4759492

**Published:** 2021-11-09

**Authors:** Hamid Reza Khorasani, Mahboubeh Sanchouli, Javad Mehrani, Davood Sabour

**Affiliations:** ^1^Department of Cancer Medicine, Cell Science Research Center, Royan Institute for Stem Cell Biology and Technology, ACECR, Isar 11, 47138-18983 Babol, Iran; ^2^Department of Stem Cells and Developmental Biology, Cell Science Research Center, Royan Institute for Stem Cell Biology and Technology, ACECR, Bani-hashem Square, 16635-148 Tehran, Iran; ^3^Department of Genetics, Faculty of Medicine, Babol University of Medical Sciences, Ganjafrooz Str, 47176-47745 Babol, Iran; ^4^Department of Periodontics, Faculty of Dentistry, Mazandaran University of Medical Sciences, Sari, Iran; ^5^Department of Tissue Engineering, School of Advanced Technologies in Medicine, Mazandaran University of Medical Sciences, Sari, Iran

## Abstract

Bone-marrow-derived mesenchymal stem cells (BM-MSCs) are one of the most widely studied postnatal stem cell populations and are considered to utilize more frequently in cell-based therapy and cancer. These types of stem cells can undergo multilineage differentiation including blood cells, cardiac cells, and osteogenic cells differentiation, thus providing an alternative source of mesenchymal stem cells (MSCs) for tissue engineering and personalized medicine. Despite the ability to reprogram human adult somatic cells to induced pluripotent stem cells (iPSCs) in culture which provided a great opportunity and opened the new door for establishing the *in vitro* disease modeling and generating an unlimited source for cell base therapy, using MSCs for regeneration purposes still have a great chance to cure diseases. In this review, we discuss the important issues in MSCs biology including the origin and functions of MSCs and their application for craniofacial and periodontal tissue regeneration, discuss the potential and clinical applications of this type of stem cells in differentiation to maxillofacial bone and cartilage *in vitro*, and address important future hopes and challenges in this field.

## 1. Introduction

Embryonic stem cells (ESCs), the pluripotent stem cells that are derived from the inner cell mass of blastocyst embryo, are able to differentiate into all body cell types as well as three embryonic germ layers, which consist of ectoderm, endoderm, and mesoderm. They are also having high proliferation and self-renewal capability. On the other hand, adult stem cells are another type of stem cells with limited differentiation capacity that are not pluripotent, but they are therefore called multipotent. Adult stem cells were only located in some organs and could differentiate into just those phenotypes found in the originating tissue [1–3]. One type of adult stem cells is MSCs that are involved in the growth, wound healing, and replacement of cells that are lost daily by exfoliation or in pathological conditions and can differentiate into several tissues including cartilage, bone, muscle, cardiac, and blood cells. MSCs are able to induce repair in neuronal, hepatic, and skeletal muscle cells after infusion in both preclinical and clinical models [4–7]. These characteristics make them a potential tool for tissue engineering and tissue repair [8]. There are some major bottlenecks for using ESCs including high tumor formation capability, which make extremely challenging to use those cells for therapeutic purposes and bring many ethical problems [9, 10]. ESCs are usually harvested from human embryos that destroy preimplantation embryos [11]. Because of ethical issues, there are limitations in using ESCs in human cell base therapy [1, 12–15]. On the other hand, those problems of using ESCs are completely excluded when men use adult stem cells or especially MSCs. MSCs are undifferentiated cells that are usually isolated from bone marrow as well as fat tissue [16]. Because of the high potential of deriving MSCs from bone marrow and an adequate number of these cells, which can easily aspirate from bone marrow, there is great demand for applying these cells for cell therapy and regenerative medicine [16–18].

Bone defects in oral and maxillofacial fractures may occur by accidents, trauma, cancer, congenital malformations, and some skeletal disease. The classical treatments of these defects are craniofacial surgery mostly on the basis of autologous bone transplantation and plastic or metal composite transplantations, which can be considered artificial bone or joints [19–23]. Conventional treatments performed to fix the defects do not regenerate lost tissues. So it is necessary to carry out other procedures to recover tissues such as root surface conditioning, bone grafting, guided tissue regeneration, and the application of growth factors, but these methods could not restore the original anatomy and physiology of tissue and also can cause some periodontal disorder. Therefore, the new therapeutic procedure of using MSCs isolated from dental tissue for regeneration has been suggested [24–28].

During the past two decades, tissue engineering and regenerative medicine have been used as new therapeutic strategies that contain biomaterials, stem cells, and tissue-inducing substances. Stem cell-based tissue engineering in tooth and periodontal regeneration particularly has been investigated after appearing of craniofacial tissue engineering in 1990, and ongoing studies are regeneration of dentin-pulp, tooth, root, whole tooth, and periodontal tissue [29, 30].

Molecular pathway and knowledge regarding MSCs proliferation and differentiation toward bone, cartilage, and teeth are still not completed. Therefore, in the context of craniofacial and periodontal tissue engineering, it is crucial to optimize differentiation protocols for stem cells toward the generation of bone, cartilage, cementum, dentin, and ligaments. The purpose of this review is to discuss recent advances in MSCs biology especially BM-MSCs and their application to regenerate and cure craniofacial and periodontal tissue regeneration, and discuss the future potential and clinical applications of this type of stem cells in differentiation to maxillofacial bone and cartilage *in vitro*, and address important future hopes and challenges in this field.

## 2. Bone-Marrow-Derived Mesenchymal Stem Cells (BM-MSCs)

MSCs are multipotent stem cells that originate from different sources of adult tissues such as adipose tissue, skin, tissues of the orofacial area, and bone marrow [31–37]. They have important potentials in regenerative medicine because of their abilities to differentiate into many types of cells. They play important role in growth, healing, replacement of cells, and repair in neuronal, hepatic, and skeletal muscle [38–40].

In recent years, it has been shown that BM-MSCs have been widely used for clinical application purposes in regenerative medicine. Therefore, the study of systemic infusion of MSCs has been useful for treating osteogenesis imperfecta. In this treatment, which was performed as a case control study by Horwitz et al., total bone mineral content and growth velocity have been significantly increased [41].

These cells are easily accessible and have the ability to generate different somatic cells as well as germ cells *in vitro* and *in vivo* [42]. In addition, BM-MSCs have certain molecular characteristics that enable to distinguish these cells from other cell types inside the bone marrow. They have specific antigenic surface proteins (markers) such as CD44, CD71, CD90, CD105, CD120a, CD124, CD166, Flt-3, and Kit ligands [43]. Cell lineage specification and differentiation of MSCs depend on the biological niche of tissue and cytokine proteins as well as specific growth factors required for MSCs differentiation. MSCs also secrete varieties of cytokines including IL-6, -7, -8, -11, -12, -14, and -15, LIF, and GM-CSF [44]. There are certain advantages of using MSCs in cell-based therapy for therapeutic purposes. Another advantage of using these cells is that MSCs have differential surface antigens compared to other mature cells in the tissue that give the possibility to use them for allogeneic transplantation. In addition to mentioned benefits of MSCs, these cells can be isolated just before cell transplantation directly from the patients. MSCs have effective activity of immunomodulatory and interaction with several types of immune cells that is crucial in generating structure for replacing damaged tissues [45]. Therefore, the risk of having cultural contamination and complication related to allogeneic immune rejection would be dramatically reduced [46].

**Figure 1:**
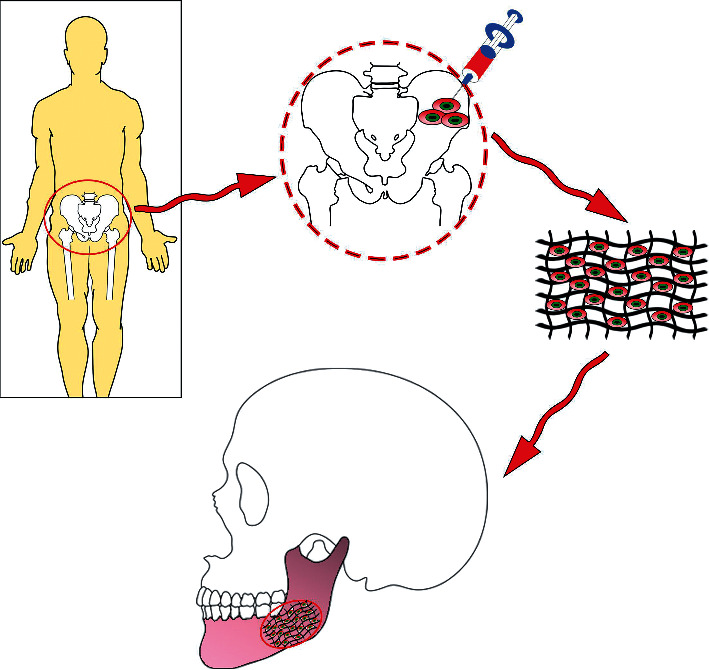
Isolation of autologous stem cells from some easy access tissues including bone marrow. There is a possibility to expand those bone marrow-derived stem cells in defined culture media on the scaffold to produce enough cells for craniofacial and dental tissue engineering purposes.

One of the major resources of stem cells that has a minimum immunogenic response for bone regeneration and osteogenesis is the isolation of autologous stem cells from some easy access tissue including fat and bone marrow [47]. There is a possibility to expand those BM-MSCs in culture to produce enough cells for craniofacial and dental tissue engineering purposes (Figure 1).

Some studies have identified important cell lines for bone regeneration and tissue engineering such as BM-MSCs, umbilical cord mesenchymal stem cells (UC-MSCs), and amniotic-fluid-derived stem cells (AFSCs). In a recent study, structure of linking recombinant human bone morphogenetic protein (rhBMP2) with silica-coated calcium hydroxyapatite (HASi) rabbit bone marrow stem cells (rBMSCs) has been shown progress in regeneration and healing bone defect. However, attributes of bone regeneration and alveolar bone defect of AFSCs have been mentioned. There is a restriction to apply that in preclinical and experimental progress of regeneration [48].

BM-MSCs and UC-MSCs are MSCs harvested from bone marrow and umbilical cords with multipotent properties that could be differentiated into osteoblasts, chondrocytes, myoblasts, adipocytes, fibroblasts, and nerve tissue. As remarkable cell lines, they have been used in craniofacial tissue engineering [11, 49–54].

UC-MSCs derived from baby's umbilical cord are younger than BM-MSCs, and because of disadvantages of BM-MSCs such as invasive process, reducing proliferation and differentiation with age, a limited amount of access, probability of destruction of the donor site, and low self-renewal, UC-MSCs could be an alternative. As regards, it is a source of stem cells and also have high plasticity and flexibility, inability to form tumors, and have low immune response, but unlike BMMSC, it cannot spontaneously differentiate into osteoblasts [55]. Some other sources of cartilage tissue engineering are mesenchymal stem cells from Wharton's jelly (WJ-MSC) and human-induced pluripotent stem cells (hiPSCs), which are multipotent and have unlimited self-renewal capability [48].

In addition, different parts of the tooth including dental pulp, exfoliated and adult teeth, apical papilla, periodontal ligament, and exofacial have generic mesenchymal cells properties [56, 57]. Dental pulp stem cells (DPSCs) extracted from adult dental pulp, stem cells from human exfoliated deciduous teeth (SHED) derived from the disposable deciduous teeth of children, stem cells from apical papilla (SCAP), and periodontal ligament stem cells (PDLSCs) extracted from discarded teeth with the potential to generate the cementum and periodontal ligament-like structure are the types of MSCs which potentially could apply for tissue engineering from dental-derived stem cells. These cells are MSCs that potentially could apply for tissue engineering from dental-derived stem cells [58–61].

In some study, capabilities of these oral stem cells in regeneration have been investigated. They proposed coculture of these cells with BM-MSCs that could augment osteogenesis capacity [10].

Taken together, the potential of MSCs to efficiently differentiate into bone, fat, muscle, dental tissues, and also cartilage as well as the noninvasive procedure to access an unlimited number of stem cells from patient's bone marrow increased the demand of using these cells for cell therapy [42, 44, 62].

## 3. Principles of Regenerative Medicine and Tissue Engineering

The field of regenerative medicine and tissue engineering is part of modern biomedicine that can cure damaged tissues and organs by using cell-based therapy. The basis of this branch of medicine is curing diseases by applying cellular, molecular, and genetics approaches. When an organ in the body is not working well, it would be an unfunctional organ, and the immediate consequence of that is generating certain diseases related to the function of that organ. In those cases, usually, only one or certain cell types do not work properly because of certain mutations or genetic alterations in cellular programming. The applications of regenerative medicine in those cases are generating proper cell types to replace them instead of defect cells in that organ. In past decades, applying stem cells for therapeutic purposes have great promise to solve this issue [63].

Friedenstein and his colleagues have firstly identified MSCs in bone marrow, which has opened a new era in regenerative medicine [64, 65]. Tissue engineering using MSCs has become one of the most interesting fields for therapeutic research [66–68]. The field of regenerative medicine by using MSCs has several advantages such as the high potential for regenerating damaged tissues without the formation of scar tissue and low risk of autoimmune rejection or disease transmission [61, 69, 70].

Regeneration of tissues is a pathway with the complicated process in three steps: inflammation, proliferation, and remodeling. During these procedures, biological signals lead to an increased amount of cells for filling wound defect and morphogenic signals that induce tissue-specific differentiation. So these procedures need components to create living tissues for replacing lost structures: scaffold, growth factors and signaling molecules, extracellular matrix, and cells [71–73]. Scaffolds are a temporary designed frame for preparing conditions for proliferation and differentiation of cells and form desired tissue. Synthetic polymers, ceramics, composites, and natural biopolymers [74–76] such as chitosan [77, 78], alginate [79], celluloses [80, 81], collagen [82–84], hyaluronan [85], fibrin [86, 87], and silk [88] are biomaterial that are used for building scaffolds. To get desired differentiation to regenerate any tissues such as dental and craniofacial bone tissues, incorporation of growth factors and related signals into the scaffold are required.

rhBMP2, 3, 4, 6, 7, and 12 are proven factors that induce osteogenesis in the craniofacial region [89, 90]. Another effective growth factor for osteogenesis is insulin-like growth factor 1 (IGF-1) that causes moving osteoblasts cells to a damaged section. Compared to other growth factors such as the basic fibroblast growth factor (bFGF) and transforming growth factor (TGF), IGF-1 is one of the best growth factors [91, 92]. Cell-binding peptide p-15, fibroblast growth factor-2 (FGF-2), growth differentiation factor 5 (GDF-5), IGF-1, matrix factors (fibronectin, amelogenins, and thrombospondin), platelet-derived growth factor (PDGF), platelet-rich plasma (PRP), vascular endothelial growth factor (VEGF), and enamel matrix derivative (EMD) are growth factors that play role in regeneration applied to dentistry [93–95]. As the third component for regeneration, stem cells can modulate chronic inflammation as an important feature in periodontitis and also can be used in periodontal tissue engineering [96].

Tumor necrosis factor (TNF) is an important cytokine in inflammation, immunity, and bone loss. TNF expression in transplanted conditioned medium (CM) obtained from cultured periodontal ligament stem cells (PDLSC-CM) and local injection of BM-MSCs as anti-inflammatory and immunomodulatory functions could be targeted for periodontitis defects repair [97, 98].

The final goal in the field of regenerative medicine and tissue engineering is differentiating stem cells into progenitor cells or fully differentiated intact cells that can replace deficient cells in those affected organs. This means that after generating differentiated cells from stem cells *in vitro*, those cells could be implanted into defective organs by direct transplantation *in vivo*. Sometimes, after transplantation, the inserted stem or progenitor cells have the potential to differentiate into target cells *in vivo* and generate the functional cells under local signaling environments and using the cellular and molecular niches more efficiently [99].

Regenerative medicine and tissue engineering are more applicable and efficient for the field of maxillofacial surgery than any other field in medicine. For instance, in the case of teeth or periodontal tissue regeneration or in maxillofacial development deficiency and hypoplasia and bone and cartilage defects or associated tumors, cleft lip, and palate, regenerative medicine is the major strategy for treatment.

## 4. Maxillofacial Reconstruction

The bone defects in the oral area include dental hard tissue defect, pulpal disease, periodontal diseases, and maxillofacial defects [100]. The most common causes of these defects are congenital malformations, accidents, trauma, cancer, and some skeletal diseases [19, 20]. Maxillofacial tissues are important because people might have low confidence with these defects [100]. The classical treatments of these defects are craniofacial surgery mostly on the base of autologous bone transplantation and plastic or metal composite transplantations, which consider as artificial bone or joints [21, 22]. Effective reconstruction or regeneration of damaged parts would be beneficial to patients both physiologically and psychologically [100]. MSCs showed promising regenerative treatment in craniofacial tissue defects. The ability of stem cells to produce several different cell types together with their widespread distribution in many adult tissues has made them an attractive target for tissue engineering applications [39]. Using MSCs for regenerating craniofacial defects may reduce the huge risk of surgery and allo- or autografting of bone or artificial composites for patients [101]. The regeneration procedure of craniofacial has been accelerated by the incorporation of DPSCs into collagen cell scaffold [102]. These dental stem cells (DSCs) are not only used for bone loss caused by periodontal diseases but also for the reconstruction of maxillofacial bones [103].

Growth factors play a crucial role in regenerating certain tissue as well as bone and cartilage. They are also required for the formation of limbs and repair of many different tissues [104, 105]. It is well known that without certain growth factors, it is not possible to differentiate cells, regenerate tissues, or repair organs. Using growth factors will help avoid spontaneous differentiation and direct differentiation into a specific lineage. Moreover, some members of the TGF-b superfamily such as BMP-2, BMP-4, BMP-6, BMP-7, BMP-12, TGF-b, PDGF, and bFGF are required for cell growth and proliferation in craniofacial regeneration [106, 107]. It has been demonstrated that some growth factors, which are required for bone formation during development, are also crucial for maxillofacial reconstruction. Some of the most important mentioned growth factors are BMP families, PDGF, bFGF, VEGF, TGF-b, and IGF [108–123]. Moreover, many research groups have reported the repair of alveolar clefts in patients with clefts using BMP proteins. In an interesting study, it has been demonstrated that in patients with congenital facial clefts, rhBMP2 autogenously can be a substitute for iliac crest bone [124, 125].

In addition, applying generating bone in older children with craniosynostosis would also be a promising procedure in maxillofacial defects. In these patients with the inability to completely remodel the skull bone after the first year of life, it is necessary to close the interpretive bone gaps within the scope of the cranioplasty by means of split graft. However, the bone supply is restricted, and there is a risk of postoperative bone holes [126–128].

## 5. Periodontal Regeneration

In order to generate cells for craniofacial and dental tissue engineering, stem cells are differentiated into chondrocytes, osteoblasts, and periodontal ligament cells. In addition, the engineering of odontoblasts and cementocytes is important and helpful for periodental regeneration [129].

The tooth is a multistructure organ that includes hard tissues such as enamel, cementum, and dentine, and pulp cavity as soft tissue [130]. The most common disease associated with teeth is periodontitis that is one of the most common and highly prevalent chronic inflammations in humans [129]. In this disease, tissues around the tooth were destroyed, resulting in tooth loss, various complications at the local and systemic level [131, 132], and injuries in the cementum, periodontal ligament, and alveolar bone [133]. The current restorations for tooth loss are dentures, including removable, fixed dentures, and dental implants [134, 135]. In the case of severe pulpitis, the capability of self-regeneration or repair is limited because the odontoblasts that produce reparative dentin are destroyed. So regeneration of the dentin-pulp complex has been investigated through the isolation and exploration of the regenerative abilities of stem cells; thus, new therapeutic possibilities may be possible. In the past decade, regenerative endodontics has gained much attention as it offers an alternative approach for the treatment of endodontically involved teeth by filling the canal with vital tissues instead of artificial materials [136].

DSCs harvested from the oral and maxillofacial region include DPSCs, SHED, PDLSCs, SCAP, and dental follicle progenitor cells (DFPCs). Dental tissue engineering is focused on dentin and pulp cavity. Odontoblasts are dentin-forming cells in dental pulp harvested from DPSCs. The periodontal ligament is another part of dental tissue as a source of PDLSCs, which are extensively investigated in bone-tissue engineering [60, 106]. The use of DSCs has effective potential in periodontal regeneration treatment. All of five types of DSCs were studied in animal models, and among them, DPSCs and PDLSCs were demonstrated in human clinical studies [137, 138].

In a study, significant improvement of the injured area of periodontal disease after the use of PDLSCs and DPSCs was shown safe effects without harmful consequences. PDLSCs can induce tissue formation around the surface of dental implants. The use of DPSCs for the regeneration of bone loss may be clinically applicable. Therefore, the use of deferent populations of DSCs in the treatment of periodontal disease can be an interesting approach [137, 139].

If the chronic inflammatory state of the supporting tissues around the teeth left untreated, it will demolish the attachment between the tooth and surrounding bone. Untreated periodontitis may eventually end up with tooth loss. Treatment strategies used to eliminate periodontitis mainly focus on the removal of dental plaque as the primary etiologic factor and long-term follow-up visits with the aim of promoting tissue repair [140]. But the problem encountered with tissue repair is incomplete regeneration of the periodontal attachment apparatus, which has been lost due to disease entity. Taken together, complete regeneration of periodontal tissues due to periodontal disease is still one of the challenging situations that need more investigation to be resolved [141]. Because of periodontitis, the periodontal attachment was destroyed as a result of inflammation and regeneration of bone and soft tissue after periodontitis remains challenging. Therefore, gene transfer approaches also have been used to deliver several growth factors that may regenerate the periodontal ligament as PDGF and BMP-7 [142, 143]. The ultimate goal for periodontal regeneration includes the reformation of new bone and cementum with the periodontal ligament at the interface of bone and cementum on the root surface of the previous disease. For this purpose, enamel matrix protein derivative (EMD) as a biological mediator plays an important role in accelerating the regeneration of periodontal tissue (cementum, alveolar bone, and attachment tissues) and stimulates the new connective tissue, cementum, periodontal ligament, and bone formation for treating the periodontal bone defects [144]. And another significant point to be considered is that using EMD and ceramic grafts (CG) in regeneration shows longer-lasting clinical results that can be safely maintained with strict supportive periodontal treatment than conservative surgery [145]. However, in conventional periodontal surgery, the use of the anorganic bovine-derived hydroxyapatite matrix/cell-binding peptide (ABM/P-15) graft has a helpful effect in a therapeutic way for periodontal regeneration as well as open flap debridement (OFD) and coronally positioned flap (CPF). But, it should be compared with other regeneration techniques, and the longevity of the materials should be studied more [146].

Generally, several methods have been used to achieve periodontal regeneration such as guided tissue regeneration, bone graft materials, growth factors, host modulating factors, and a combination of the mentioned modalities. Although few reports showed success in periodontal regeneration by using these techniques, the success rate in challenging clinical situations is below the expectation [25].

Periodontal regeneration began with the migration of progenitor periodontal ligament cells over the denuded root surface, then continued by attaching to the root surface. Afterward, connective tissue was generated and inserted into the newly formed cementum. On the other hand, bone progenitor cells were migrated along with regeneration of periodontal ligament [147].

Successful regeneration of the diseased periodontium requires the coordination and availability of three key regulators of regeneration including scaffolds, signaling molecules, and cells [148].

For the first key, various natural and synthetically materials are available for use in a scaffold structure for tissue engineering and regenerative aims. Among them, chitosan has been found favorable for tissue regeneration to produce bioactive scaffolds that accelerate osteoblast proliferation and bone formation. The incorporation of chitosan with natural polymers such as collagen and silk fibroin has also been detected as an accelerator in the proliferation of osteoblasts and mesenchymal cells in vitro. Recently, shitosan-collagen fibrous membranes were used in periodontal and bone regeneration, and it was evaluated as a proper scaffold for human periodontal ligament cells (hPDLCs) to be more adhesive and capable in growth with no significant sign of reaction and inflammation *in vivo* studies [149].

Furthermore, different types of cells have been studied as a good therapeutic option for periodental regeneration. Recent studies showed that dental-derived stem cells have great potential for the regeneration of periodontal tissues around the teeth. Dental-derived stem cells include PDLSCs and dental socket stem cells (DSSCs) that may have promising usages in the future for periodontal regeneration [150]. There are some other sources of dental-derived stem cells that are not enough attractive to focus and use for periodontal regeneration including DPSCs because of their low capacity to form cementum and lower attachment ability compared to PDLSCs [151, 152] and stem cells of SHED, dental follicle, and dental apical papilla because of limited scientific evidence [153–155]. Here, we describe how far some of these MSCs can be helpful for periodental regeneration.

## 6. Periodontal Ligament Stem Cells (PDLSCs)

Among all the MSCs, PDLSCs are the main candidate stem cells in periodontal regeneration. Transplanting PDLSCs directly into periodontal defect areas resulted in periodontal regeneration [156].

PDLSCs exhibit self-renewal ability and express cell surface markers similar to MSCs derived from bone marrow. They are able to differentiate toward osteoblasts, odontoblasts, adipocytes, neural cells, cementoblasts, and chondroblasts *in vitro* [157–161] that have an important role in creating the periodontal tissues as a protective supporting unit covering the teeth [162]. In addition, PDLSCs from extracted teeth can recently culture, expand, and differentiate *in vitro* and thus may have important applications in achieving efficient dental tissue regeneration. Stem cells from the periodontal ligament express cell markers such as CD44, CD73, CD90, CD105, CD106, and CD146, but they do not possess hematopoietic markers such as CD31, CD34, and CD45 [163–165]. PDLSCs like other sources of DSCs have the potential to transform into adipogenic, osteogenic, and chondrogenic cells *in vitro* [148, 166]. In *in vivo* situations when these cells were implanted subcutaneously in immune-compromised mice, they had the potential of differentiating to functional cementoblasts to form collagen fibers embedded in cementum-like tissue. This property could indicate the potential of these cells in creating the periodontal ligament complex. Furthermore, when these cells were transplanted into periodontal defects that were generated in immune-compromised mice, PDLSCs regenerate periodontal ligament-like tissue that is also associated with the trabecular bone in the regenerated periodontal tissue [157].

In regenerative medicine, a novel study of transferring human PDLSCs onto decellularized amniotic membrane as a scaffold surface and transplanting into the periodontal defect in the rat has been performed by Iwasaki's group. They have demonstrated that this process improves periodontal defect in comparison to transplant amnion only by examining the formation of cementum, periodontal ligament, and bone with microcomputed tomography and histological observation, and also transplanted cells were traced by using PKH26 and human Alu sequence detection by PCR [167].

Torii et al. analyzed gene expression as well as the mineralization potential of primary and immortal hPDLCs and their immunophenotype in order to study the cementogenic potential of PDLSCs purified from the human periodontal ligament. Mineralization induction medium contained b-glycerophosphate, ascorbic acid, and dexamethasone; both normal hPDLCs and immortal cells showed higher levels of mineralization compared with cells grown in the normal growth medium. Both cell types were positive for CD44, CD73, CD90, and CD105. They were also positive for stage-specific embryonic antigen-3 (SSEA-3), which is a multipotential stem cell marker. Moreover, culturing the PDLCs with rhBMP-2 or rhBMP-7 led to an expression of cementum attachment protein and cementum protein. The above results confirmed the existence of multipotential MSCs with the cementogenic potential in normal and immortal hPDLCs [168].

In periodental tissue engineering, PDLSCs as a great source of tooth tissue could increase cell expansion. Zhang et al. have recently shown that the combined application of FGF-2 and A83-01 remarkably is effective in the promotion of PDLSCs biological behavior in cell expansion, reduction of cell apoptosis, and also increasing of proliferation, stemness expression, paracrine action, later osteogenic differentiation, and mineralization of PDLSCs [133].

Yu et al. investigated the maxillary sinus augmentation in the canine model. They studied the combination of Bio-Oss® bone material with tissue-engineered bone derived from PDLSCs, and BM-MSCs were augmented with bilateral maxillary sinus floor augmentations in six beagles randomly. First, they were labeled with fluorescent probes for 12 weeks. Then, they were analyzed for new bone deposition, mineralization, and remodeling in the augmented area using maxillofacial computed tomography, scanning electron microscopy, and histologic and histomorphometric analyses. The osteogenic potential of group A was smaller than two other groups. In group C, the level was higher than group B; however, the difference was not statistically relevant. Their conclusions were about the promotion of bone formation and mineralization and maintenance of the total increase in volume of the maxillary sinus via incorporating PDLSCs or BM-MSCs onto Bio-Oss® as promising candidates for maxillary sinus augmentation [169]. Altogether, the unique features of PDLSC could be a cell-based regenerative periodontal therapy in the near future as effective and motivating cell origin.

## 7. Future Perspective

In recent years' tremendous amount of progress has been made in the field of stem cells (either in ESCs or iPSCs) mostly in animal models, but the main focus of research on stem cells in humans is *in vitro* studies, which are significantly valuable. Although transplantation of animal model stem cells or differentiated cells in rodents especially in mice is very usual, performing similar experiments in humans because of the ethical issue is not possible. Therefore, researchers mostly rely on *in vivo* experiments to study human stem cells and the differentiation of different lineage from these cell types. The only stem cells that are very popular to use for *in vivo* studies and clinical applications in humans are MSCs especially BM-MSCs. Access to these cells from humans is quite easy. They have a low immunogenicity risk for transplantation as well as there is no risk of tumor formation in the recipient. All of those advantages made MSCs a favorite stem cells model for scientists because there are no ethical concerns for them. On the other hand, the differentiation protocols of bone, cartilage, or dental cells from MSCs are optimizing very quickly, and together by using biomaterials and growth factors in defined culture media, there is great hope that autograft and allograft transplantation of MSCs become widely utilized in the clinic for therapeutic purposes.

Due to the associated aesthetic characteristics of craniofacial tissue and the psychological importance of facial beauty in human societies, this region requires the highest consideration in tissue engineering. For the successful reconstruction and regeneration of maxillofacial and periodontal complex tissue structures and the restoration of aesthetic characteristics, not only BM-MSCs and growth factors are essential, but also numerous biomaterials and scaffolds should be utilized.

Applying stem cells has great potential in dentistry and maxillofacial rehabilitation since it could provide better cellular niches for the regeneration of defect tissue. Stem cell therapy plays a crucial role in the field of dentistry and maxillofacial reconstruction. With recent advancements in the field of tissue engineering and cell therapy, clinical challenges related to maxillofacial reconstruction can be solved. One of the main challenges of tissue engineering in clinical treatment is to reduce invasive surgical procedures by applying cellular processes and biological materials or artificial components cultivated from the patient's own cells. In conclusion, recent advances in using 3D printing of composite tissue with the complex structure for generating different tissues together with promising broad potential of MSCs from different sources especially BM-MSCs could pave the ways for scientists to improve the methods for oral maxillofacial surgery, plastic surgery, and cure craniofacial anomalies.

## Data Availability

Data are available upon request to the corresponding author.
